# Atrial arrhythmias and acute pericarditis triggered by bleach ingestion-associated oesophageal perforation: a case report

**DOI:** 10.1093/ehjcr/ytae046

**Published:** 2024-01-30

**Authors:** Ahmed Saleh, Alex D'amico, Ammar Hasnie, Stephen Clarkson, Brittain Heindl

**Affiliations:** Internal Medicine, University of Alabama at Birmingham School of Arts and Humanities: The University of Alabama at Birmingham College of Arts and Sciences, 1802 6th Ave S, Birmingham, AL 35233, USA; Internal Medicine, University of Alabama at Birmingham School of Arts and Humanities: The University of Alabama at Birmingham College of Arts and Sciences, 1802 6th Ave S, Birmingham, AL 35233, USA; Internal Medicine, University of Alabama at Birmingham School of Arts and Humanities: The University of Alabama at Birmingham College of Arts and Sciences, 1802 6th Ave S, Birmingham, AL 35233, USA; Internal Medicine, University of Alabama at Birmingham School of Arts and Humanities: The University of Alabama at Birmingham College of Arts and Sciences, 1802 6th Ave S, Birmingham, AL 35233, USA; Internal Medicine, University of Alabama at Birmingham School of Arts and Humanities: The University of Alabama at Birmingham College of Arts and Sciences, 1802 6th Ave S, Birmingham, AL 35233, USA

**Keywords:** Oesophageal perforation, Bleach ingestion, Acute pericarditis, Atrial arrhythmia, Atrial fibrillation, Atrial flutter, Case report

## Abstract

**Background:**

Acute pericarditis due to oesophageal perforation and caustic injury is a rare presentation of bleach ingestion. Cardiac arrhythmias such as atrial fibrillation and atrial flutter have been associated with certain aetiologies of acute pericarditis. This case report presents a unique occurrence of acute pericarditis following bleach ingestion and intermittent atrial fibrillation and atrial flutter triggered by liquid intake.

**Case summary:**

A 36-year-old male with no significant past medical history presented after attempted suicide by ingesting bleach. He had acute pericarditis resulting from caustic oesophageal perforation and extensive mediastinal injury. In the following days, he developed recurrent episodes of atrial fibrillation and atrial flutter following fluid intake, prompting treatment with metoprolol. On Day 5 of hospitalization, he underwent an oesophagogram and developed persistent atrial arrhythmia with haemodynamic instability requiring cardioversion. He underwent thoracoscopic surgery to address the oesophageal injury. A jejunostomy tube was placed and he had complete resolution of his recurrent atrial arrhythmia.

**Discussion:**

This case highlights a rare presentation of atrial arrhythmias and acute pericarditis caused by corrosive oesophageal injury due to bleach ingestion. The effective management of such cases necessitates a co-ordinated approach, involving the collaboration of cardiothoracic surgeons, cardiologists, and critical care specialists, with the aim of enhancing patient outcomes and mitigating the life-threatening risks associated with oesophageal perforation and cardiac arrhythmias. Furthermore, this case underscores the imperative for further research to better understand the relationship between traumatic acute pericarditis and atrial arrhythmias, offering the potential for improved patient care in these intricate clinical scenarios.

**Learning points**

- Oesophageal perforation can result from various causes, including trauma, caustic injury, and iatrogenic factors.
- Bleach ingestion is a common cause of severe gastroesophageal burn injuries, pulmonary toxicity, oesophageal perforation, and mediastinitis.
- Acute pericarditis is a rare complication of caustic mediastinal injury, and its association with tachyarrhythmias is not well-understood.
- Water-soluble contrast studies may be used to diagnose oesophageal leaks but can contribute to mediastinitis.

## Introduction

Oesophageal perforation is a rare but potentially serious condition that can result from various causes, including trauma, hyperemesis, malignancy, foreign bodies, caustic injury, or iatrogenic factors.^1^ While mediastinitis and peritonitis are known complications of oesophageal perforation from bleach ingestion, acute pericarditis and subsequent extravasation inducing cardiac arrhythmias have not been previously reported. This case report describes a unique instance of acute pericarditis following bleach ingestion, along with recurrent atrial fibrillation and atrial flutter induced by liquid ingestion.

## Summary figure

**Table:** 

Timepoints	Clinical descriptions
Admission	Presents from jail in acute distress and haemodynamic compromise after ingestion of bleach
Hours	Stabilized requiring intubation, vasopressor support, and broad-spectrum antibiotics; bilateral chest tubes placed to suction
Days 2–4	Extubated to nasal cannula and transitioned off vasopressor support; noted to have multiple episodes of atrial arrhythmia after swallowing liquids
Day 5	Underwent barium swallow study and developed acute atrial arrhythmia with haemodynamic instability shortly after administration of contrast
Day 6	Underwent a left-sided video-assisted thoracoscopic surgery for pleural debridement, mediastinal drainage via a cervical incision, endoscopy, and nasoenteric feeding tube insertion
Days 6–61	Hospital stay was extended for ongoing infection and effusion, requiring an extended chest tube placement. He was discharged for clinic follow-up within a month, free of recurrent arrhythmia

## Case presentation

**Figure 1:**
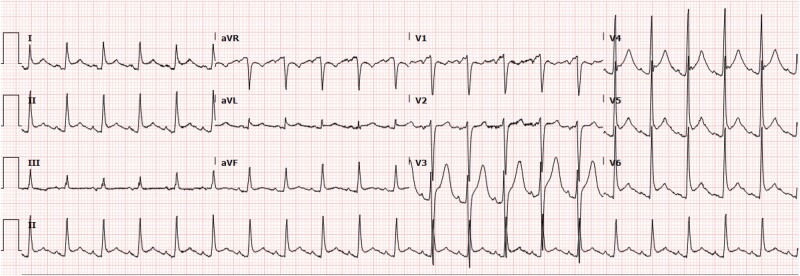
Initial electrocardiogram (EKG) on presentation to the emergency department. EKG is concerning for Spodick’s sign with downward slopping in leads I and II. Diffuse PR depressions can be appreciated.

A 36-year-old male, without significant medical history, presented to our emergency department in severe respiratory distress after a confirmed suicide attempt involving bleach ingestion. On arrival, his blood pressure was 70/50 mmHg, oxygen saturation was 90% with a non-rebreather mask, and his respiratory rate was 40 breaths per minute. His examination revealed bilateral lung base crackles, distant heart sounds with regular tachycardia, pleuritic friction rub, and crepitus upon anterior neck palpation. Due to his critical condition, immediate intubation and haemodynamic support with norepinephrine and vasopressin were required. An initial 12-lead ECG showed sinus tachycardia with PR depressions and downward sloping TP segments in specific leads (Spodick’s sign), suggesting acute pericarditis (*Figure 1*).^2^ A subsequent transthoracic echocardiogram showed a trace pericardial effusion with preserved left ventricular function and no acute valvular abnormalities.

**Figure 2:**
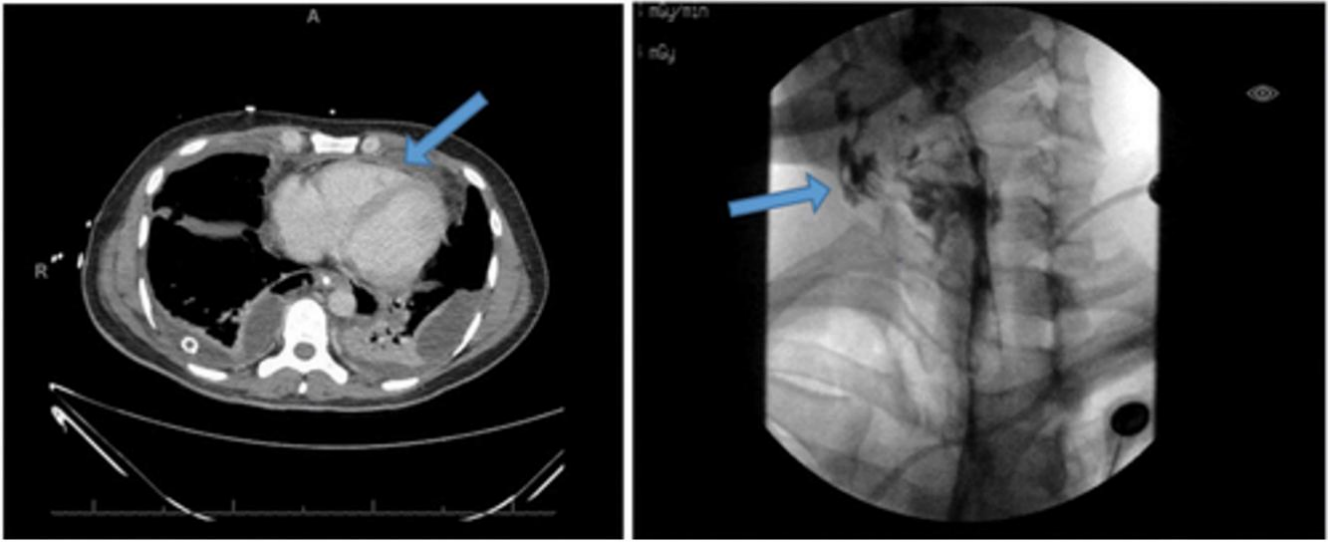
(*A*) CT chest revealing for bilateral effusion of the posterior lung fields and trace effusion of the anterior mediastinum (arrow) surrounding the pericardium, (*B*) still imaging from oesophagram prior to development of tachyarrhythmia with extravasated fluid visualized within the mediastinum (arrow).

His initial lab results showed a high-sensitivity troponin of 65 ng/L, a white cell count of 29 × 10^3^ cmm (normal range: 4–11 × 10^3^ cmm), lactic acid level of 3.3 mmol/L (normal range: 0.5–2.2 mmol/L), an anion gap of 17, and a serum creatinine of 2.6 mg/dL, compared to a baseline of 1 mg/dL (*Table 1*). Computed tomography revealed a defect in the right lateral oropharyngeal wall, with gas and contrast material tracking into the retropharyngeal space, mediastinum, bilateral pleural spaces, and a small pericardial effusion (*Figure 2*). Thoracic surgery was consulted, and bilateral chest tubes were placed with suction. Pleural fluid cultures grew extended-spectrum beta-lactamase *Klebsiella pneumoniae*. The patient received ceftolozane–tazobactam infusion. On the second day of hospitalization, his blood pressure and respiratory status improved, and he was weaned off vasopressors and extubated to nasal cannula.

**Table 1:** Laboratory values from the time of admission until the development of tachyarrhythmia

Blood	Date				
	Day 5	Day 4	Day 3	Day 2	Day 1
Lactic acid (mmol/L) (0.5–2.2 mmol/L)				2.0	3.3
Creatinine (mg/dL) (0.6–1.3)	1	1.3	1.3	1.9 H	2.5 H
Magnesium (mg/dL) (1.6–2.6)	2.1	2.6 H	—	4.2 H	4.7 H
Adjusted calcium (mg/dL) (8.5–10.5)	8.7	8.7	8.3 L	8.2 L	8.5
Protein (g/dL) (6.0–8.3)	—	4.4 L	5.2 L	4.7 L	4.8 L
Albumin (g/dL) (3.4–5.4)	—	1.7 L	2.1 L	2.1 L	2.3 L
Phosphate (mg/dL) (2.5–4.5)	1.7 L	1.7 L	—	2.1 L	2.3 L
Sodium (mEq/L) (133–145)	152 H	160 H	156 H	148 H	—
Potassium (mEq/L) (3.5–5.1)	4.1	4.2	3.85	3.7	—
Chloride (mEq/L) (98–107)	122 H	127 H	122 H	112 H	7.1 L
Bicarb (mEq/L) (22–28)	21 L	21 L	21 L	21 L	8.5
Glucose (mg/dL) (70–100)	97	139	120	—	—
WBC (10^3^/cmm) (4.5–11)	13.75	13.73	14.74	21.71	31.26
Hgb (g/dL) (13.5–17)	14	13.1	14.3	12.8	13.5
Hct (%) (39–50)	42	39	42	38	40
Platelets (10^3^/cmm)	140.8	103.9	103.3	90.7	92.2

**Figure 3:**
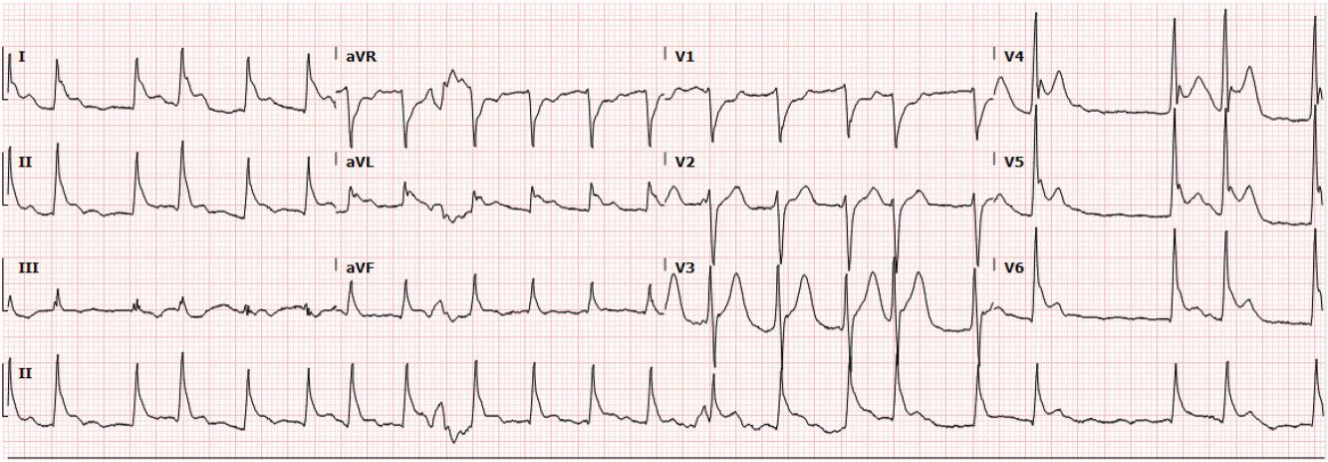
Electrocardiogram (EKG) captured at the time of acute atrial fibrillation following ingestion of liquids.

In the following days, the patient experienced three episodes of acute atrial fibrillation and atrial flutter immediately after drinking small amounts of fluids. These arrhythmias were either self-limited or responded to a 5 mg intravenous metoprolol dose in one instance. On the fifth day of hospitalization, a barium swallow study was performed to evaluate oesophageal perforation, revealing a large pharyngeal leak extending into the mediastinum adjacent to the proximal oesophagus. Immediately after image acquisition, the patient developed sustained atrial flutter with a heart rate of 150 b.p.m. and a mean arterial pressure of 55. He was treated with intravenous metoprolol tartrate and amiodarone, but the arrhythmia persisted, leading to worsening haemodynamic compromise. Ultimately, synchronized cardioversion with 200 J successfully restored sinus rhythm (*Figure 3*).

The following day, he underwent left-sided video-assisted thoracoscopic surgery, which included debridement of the pleural space, drainage of the anterior and posterior-superior mediastinum through a cervical incision, flexible endoscopy, and nasoenteric feed tube placement. Due to the severe consequences of drinking, a jejunostomy tube was placed, effectively preventing further arrhythmias. The rest of his hospitalization was extended due to persistent infection and recurrent effusion, necessitating prolonged chest tube placement. He remained free from arrhythmia thought this time and antiarrhythmic medication was discontinued. He was discharged on the 61st hospital day with close follow-up with the thoracic surgery team. Roughly 1 month following discharge, he appeared for a follow-up appointment in outpatient clinic. There was no evidence of arrhythmia at this time and an EKG was obtained which demonstrated normal sinus rhythm.

## Discussion

This case report highlights a cause of atrial fibrillation and atrial flutter resulting from oesophageal leakage leading to acute pericarditis and recurrent pericardial irritation upon fluid ingestion. Supraventricular tachycardia is common in the context of sepsis and acute illness. However, this patient’s tachyarrhythmias occurred days after he was stabilized, and sepsis had resolved. The large oesophageal leak and subsequent recurrent fluid extravasation during drinking are suspected as the cause of his arrhythmias, given their immediate onset following fluid intake and their resolution after jejunostomy tube placement. This case report emphasizes three key points: bleach ingestion as a cause of gastroesophageal perforation and pericarditis, the association between pericardial irritation and tachyarrhythmias, and the treatment of such patients. Addressing these points aims to enhance our understanding and improve patient care.

Bleach ingestion leads to a substantial number of intensive care unit admissions, with ∼45 000 cases reported annually.^3^ Sodium hypochlorite, the active ingredient in household bleach, is known for its corrosive properties. When combined with water, it forms highly reactive hypochlorous acid, conferring potent antibacterial and antifungal properties. This highly toxic substance can cause severe gastroesophageal burn injuries, pulmonary toxicity, and mediastinitis, especially when associated with oesophageal perforation and leakage.^4,5^ Sodium hydrochlorite ingestion as a cause of acute pericarditis is rare, as it requires extensive injury to disrupt the fibrous pericardium and sustain such a complication.

Acute pericarditis is diagnosed by identifying at least two of the following four criteria according to current European Society of Cardiology guidelines: pleuritic pericardial chest pain, which worsens with lying down and improves with leaning forward; pericardial friction rub; ECG changes, including diffuse ST-segment elevation and/or PR depression; and new or worsening pericardial effusion.^6^ The relationship between acute pericarditis and atrial arrhythmias remains controversial. One study by Imazio *et al*.^7^ identified an incidence of atrial fibrillation and atrial flutter in patients meeting acute pericarditis criteria of ∼4%, similar to age-matched populations without pericarditis. Other studies suggest that the incidence of arrhythmias in acute pericarditis varies widely depending on the underlying cause, reaching as high as 25% in some cases.^8,9^ As the aetiology of acute pericarditis can be diverse (infectious, traumatic, autoimmune, and etc.), the association between tachyarrhythmias and traumatic acute pericarditis due to caustic injury remains inadequately quantified. Moreover, the mechanism behind the induction of atrial arrhythmias by recurrent pericardial irritation has not been previously explored.

## Conclusion

This case report highlights an unusual complication associated with oesophageal perforation. Atrial arrhythmias in the context of traumatic acute pericarditis due to caustic injury are rare and inadequately documented in the literature. Clinicians should be aware of potential factors contributing to tachyarrhythmias in critically ill patients with oesophageal perforations. Prompt diagnosis and appropriate management are critical in optimizing patient outcomes in these challenging cases.

## Data Availability

No new data were generated or analysed in support of this research.

## Lead author biography

Ahmed Saleh is a PGY-2 internal medicine resident, aspires to a career in cardiology, particularly electrophysiology. His passion for enhancing patient care and outcomes is evident through his research pursuits in epidemiology, preventative cardiology, and equitable healthcare access for underserved populations. With a commitment to learning and a vision for transforming cardiovascular medicine, Ahmed is poised to make a profound impact in the field.

**Consent:** Consent for publication has been obtained, in line with the COPE best practice guidelines, and the individual who is being reported on is aware of the possible consequences of that reporting.

**Funding:** No funding for this research project.
